# Exploring the Relationship between Experiential Avoidance, Coping Functions and the Recency and Frequency of Self-Harm

**DOI:** 10.1371/journal.pone.0159854

**Published:** 2016-07-21

**Authors:** Emma Nielsen, Kapil Sayal, Ellen Townsend

**Affiliations:** 1 School of Psychology, University of Nottingham, Nottingham, United Kingdom; 2 Division of Psychiatry and Applied Psychology, School of Medicine, University of Nottingham, Nottingham, United Kingdom; Central Institute of Mental Health, GERMANY

## Abstract

This study investigated the relationship between experiential avoidance, coping and the recency and frequency of self-harm, in a community sample (*N* = 1332, aged 16–69 years). Participants completed online, self-report measures assessing self-harm, momentary affect, experiential avoidance and coping in response to a recent stressor. Participants who had self-harmed reported significantly higher levels of experiential avoidance and avoidance coping, as well as lower levels of approach, reappraisal and emotional regulation coping, than those with no self-harm history. Moreover, more recent self-harm was associated with lower endorsement of approach, reappraisal and emotion regulation coping, and also higher levels of both avoidance coping and experiential avoidance. Higher experiential avoidance and avoidance coping also predicted increased lifetime frequency of self-harm. Conversely, increased approach and reappraisal coping were associated with a decreased likelihood of high frequency self-harm. Although some of the effects were small, particularly in relation to lifetime frequency of self-harm, overall our results suggest that experiential avoidance tendency may be an important psychological factor underpinning self-harm, regardless of suicidal intent (e.g. including mixed intent, suicidal intent, ambivalence), which is not accounted for in existing models of self-harm.

## Introduction

Self-harm, defined as “self-injury or self-poisoning irrespective of the apparent purpose of the act” [1], is a major public health concern [2]. As well as being indicative of serious psychological distress [3], self-harm is related to an increased rate of attempted suicide and death by suicide [4].

Self-harm is highly prevalent, with onset typically occurring in adolescence [5,6]. Given the high risk of repetition [7] and negative outcomes associated with escalation of these behaviours, it is important to explore the dynamics underlying self-harm, and in particular what might drive and break cyclical re-engagement in this behaviour. Experiential avoidance has been proposed as a central component in this [8].

### Experiential avoidance and coping

In the broadest sense, the term experiential avoidance (EA) encompasses all forms of avoidance and escape behaviour that are utilised as methods to alter the form, intensity and/or function of experience; EA is defined by the drive to avoid distress [9]. The term denotes a desire to suppress unwanted emotions, thoughts and sensations and relates to an individual’s response to their feelings, rather than what they are feeling per se [9,10]. Experiential avoidance is characterised by two composite aspects (a) an unwillingness to remain in contact with private, aversive experiences, and (b) those actions taken in order to alter aversive experience(s) and/or the events that precipitate them [11].

While some have outlined EA as a discrete strategy of emotion regulation [12], in its original conceptualisation [11] experiential avoidance is considered a style of interacting with private events. That is, a generalised tendency which may, secondarily, increase the likelihood of engaging in particular coping strategies that serve similar functions (e.g., thought suppression, behavioural avoidance). Therefore, while coping and experiential avoidance overlap, the construct of EA appears to have arisen from an awareness of the importance of interoceptive exposure [13] (i.e., internal experiences/sensations).

Experiential avoidance has been outlined as a central component in the development of increased perceived stress due to its relationship with coping dynamics; emotional avoidance demonstrates a propensity to engender coping responses that diminish resources, thus reducing an individual’s capacity to deal with day-to-day activities and tasks [13]. As highlighted by Kashdan et al. [14] “prolonged, inflexible non-acceptance of emotional responses can consume attention, vitality and other resources, leaving fewer resources to cope and thrive in everyday life” (p.437). Conversely, flexible, responsive coping and a tendency towards emotional acceptance are less of a burden on cognitive resources. Thus, individuals with reduced reliance on experiential avoidance often report lower levels of perceived stress and demonstrate coping responses that allow them to navigate the nuances of life with relative ease [15]. Accordingly, individuals who exhibit low experiential avoidance report higher quality of life [13].

Therefore, while experiential avoidance may be an effective way of terminating unwanted emotional states in the short-term, pervasive reliance has detrimental consequences and can lead to a severely constricted life [8]. Experiential avoidance becomes a disordered process when applied rigidly and habitually and/or when excessive energy is directed to the control and management of unwanted private experience, to the detriment of the individual.

### The Experiential Avoidance Model (EAM) of self-harm

Although it is argued that self-harm serves multiple functions simultaneously, the reasons most consistently endorsed in both clinical and theoretical literature relate to the avoidance and elimination of, or escape from, aversive internal experience [16–18]. Preliminary empirical data provides support for a strong experiential avoidance component [19]. To this end, engagement in self-harm behaviours can be conceptualised as a specific manifestation of a broader experientially avoidant coping function: a means of gaining control over an otherwise uncontrollable stress response [8].

The experiential avoidance model (EAM) [8] provides a theoretical framework to understand the factors which may control engagement in self-harm. The model proposes that self-harm is principally a manifestation of emotional avoidance, although the behaviour may also serve to allow an individual to avoid alternate internal experience (e.g., thoughts, memories, somatic sensations). EAM views self-harm as a coping strategy used to modulate unwanted or intolerable aversive mental states and posits that a stimulus that elicits an intense aversive emotion may be conducive to a shift towards avoidance repertoires, such as self-harm. Engaging in self-harm leads to short-term symptom relief. This resultant reduction in distress serves to maintain and strengthen the reliance on self-destructive behaviours; the avoidant behaviour is reinforced via temporary relief from, or elimination of, the internal state and forms a self-perpetuating cycle. Thus, the avoidant behaviour becomes an automatic escape response.

Research has begun to delineate factors which may be associated with an increased propensity to adopt experientially avoidant strategies. Avoidant response tendencies are especially likely in individuals experiencing heightened emotional intensity, those with deficits in arousal regulation or those who have poor distress tolerance [8]. Additionally, individuals who have difficulty implementing alternative coping strategies, when under conditions of emotional arousal, are also at risk [8]. The deficits outlined increase the likelihood of encountering difficulty thinking, planning or implementing more functional coping approaches. With diminished resources, individuals may demonstrate a preference for quick, easily executable strategies such as self-harm behaviours, to mitigate aversive emotional experiences. These strategies are likely to be pronounced in those high in impulsivity [8].

These factors may be important in developing an understanding of the continuation/ exacerbation of self-harm, as well as an ability to discern these individuals from those who stop, or who have engaged in self-harm less recently or frequently. This has implications for intervention planning.

### Coping functions

Both coping responses and coping strategies refer to intentional behavioural and cognition coping efforts, employed in response to a specific stressor [20]. Trait models group behaviour and cognitions with a common mode of action into coping styles [21]. Theoretical accounts posit that these personality orientations predict responding to stressors across both context and time. However research evidence questions such dispositional conceptualisations [22,23]. Rather, empirical evidence supports a transactional approach to coping [22,24]. Such ‘process perspectives’ encompass notions of change, defining coping as evolving behavioural and cognitive actions to deal with both the internal and situational demands an individual encounters.

Functional accounts of coping are distinguishable from coping style accounts by a shift in emphasis from what the individual does, to what these behavioural and/or cognitive efforts do (psychologically) for that individual [21,25]. That is to say, while accounts of coping style seek to group coping responses with similar modes of action, coping functions seek to describe individuals’ self-defined coping goals. Accordingly, the assessment of coping styles provides a structural account of coping, whereas eliciting functional accounts allows the individual to outline what they believe their coping response(s) (e.g., cognitions, behaviours) will achieve. Importantly, in structural accounts, the meaning ascribed to coping responses is inferred by researchers. Proponents of functional approaches posit that coping functions are transactionally defined, therefore a posteriori inference is problematic. For example, coping of a similar style may be functionally different; social support seeking may be categorised as adaptive (e.g., if considered to serve problem-solving functions, or facilitate approach and reappraisal) or maladaptive (e.g., if considered functionally avoidant) [13]. Indeed, researchers may have difficulty grouping coping styles into functional codings [26]. Further, one coping strategy can serve more than one coping function [21]. Therefore, the current study seeks to assess coping function, rather than infer functionality.

Divergent transactional accounts differ in terms of number of functional dimensions conceptualised. The most parsimonious accounts consider all coping functions subsumed under a single uni-dimensional construct, approach- avoidance [27]. More complex structures consider multiple functional dimensions, e.g., emotion focused vs. problem focused [28,29] (see Table 1). Following an extensive review of extant coping measures, Cox and Ferguson [25] posit four qualitatively distinct coping functions: emotional regulation (dealing with the emotional consequences of a problem), approach functions (dealing directly with the problem), reappraisal (readdressing and reinterpreting the meaning of a situation) and avoidance (allowing the individual to ignore the existence of the situation). The relationship between these functional dynamics and more parsimonious accounts are summarised in Table 1.

**Table 1:** The relationships between the four functional coping dynamics outlined by Cox and Ferguson [25] and alternative conceptualisations of functional coping. Table adapted from Ferguson and Cox [21].

Functional coping dimensions	Description	Authors
***Unidimensional***		
Approach- avoidance	(A single dimension subsuming approach, avoidance, reappraisal and emotional regulation dimensions)	[27]
***Two dimensions***		
Approach vs. avoidance	Separate dimensions for: Approach coping (approach, reappraisal and emotional regulation dimensions) Avoidance coping (avoidance)	[32] [33] [34]
Emotion focused vs. problem focused	Version A—Separate dimensions for: Problem focused coping (approach, reappraisal and avoidance dimensions) Emotion focused coping (emotional regulation)	[28]
	Version B—Separate dimensions for: Problem focused coping (approach and reappraisal dimensions) Emotion focused coping (emotional regulation and avoidance dimensions)	[29]
***Three dimensions***		
Problem focused vs. emotion focused vs. reappraisal	Version A—Separate dimensions for: Problem focused coping (approach) Emotion focused coping (emotional regulation and avoidance dimensions) Reappraisal coping (reappraisal)	[29]
	Version B—Separate dimensions for: Problem focused coping (approach and avoidance dimensions) Emotion focused coping (emotional regulation) Reappraisal coping (reappraisal)	[29] [28]
	Version C—Separate dimensions for: Problem focused coping (approach) Emotion focused coping (emotional regulation) Reappraisal (Reappraisal and avoidance dimensions)	[29] [35]

Research indicates that coping and experiential avoidance are overlapping, but discrete, constructs [13]. Avoidance coping strategies, or the propensity to engage in behavioural avoidance (e.g., turning to work) as a means to down-regulate an affective response in stressful situations may be considered a component processes of experiential avoidance (10, 30). However, experiential avoidance is a broader construct incorporating additional coping facets, such as aspects of detachment coping and emotion suppression [30] as well as a generalised tendency for decreased psychological flexibility [11], heightened cognitive entanglement with internal experiences and increased perceived uncontrollability of threats [30]. Thus, experiential avoidance encompasses an emphasis on internally focused events and refers to both the cognitive (e.g., attention, appraisal) and behavioural (e.g., avoidant coping) strategies utilised as a means of altering aversive experiences and the events that elicit them [11]. Research exploring the underlying factor structure of coping and experiential avoidance indicates that experiential avoidance relates to both avoidant coping and emotion-focused coping that is not typically considered avoidant (e.g., self-blame, emotional support seeking etc.). Importantly, those with heightened experiential avoidance tendencies not only suppress aversive emotion, but may also process and express affect maladaptively. This may account for the unique variance in explaining psychological distress, health and wellbeing contributed by experiential avoidance, beyond coping [13].

The present study focuses on experiential avoidance and the implementation of coping strategies, specifically the perceived functional dynamics of elected coping responses. While both structural and functional accounts are paramount to building a comprehensive understanding of coping, there is a lack of research addressing functional coping dynamics in relation to self-harm. Indeed—despite an increased understanding that functionality is paramount in understand the complex and dynamic nature of self-harm [31]—to the best of our knowledge, this is the first study which addresses functional coping in relation to both life-time self-harm and self-harm recency.

### Current study

The EAM [8] proposes that the tendency towards emotional avoidance is particularly strong in individuals who self-harm. While empirical evidence supports this notion of between-group differences in aspects of emotional responding where the self-harm group is comprised exclusively of those with recent behaviour engagement, there is a dearth of evidence examining the proposed relationship in more diverse samples [36]. The present study seeks to extend the understanding of the relationship between experiential avoidance, coping function and self-harm by exploring both lifetime engagement and the recency of self-harm. In line with the theoretical account, it is hypothesised that those with a lifetime history of self-harm will report higher levels of experiential avoidance and higher levels of coping perceived as avoidant and emotion regulatory in function, than those who have never self-harmed. Further to this, it is hypothesised that increased endorsement of experiential avoidance and higher levels of both avoidant and emotional regulatory coping will predict self-harm recency.

Given that the EAM [8] is a behavioural model, frequency, as well as recency of engagement, is hypothesised as being a key characteristic when considering the relationship between self-harm, experiential avoidance and coping function. The model posits that with successive reductions in negative affect associations are strengthened, such that self-harm becomes an automatic escape response. Thus, individuals become progressively reliant on emotionally avoidant repertoires. Those with higher lifetime frequency of behaviour engagement will have been exposed to a greater number of experiences or ‘trials’, thus it is proposed that the association will be stronger. Therefore it is hypothesised that higher frequency self-harm will be associated with increased endorsement of experiential avoidance and higher levels of both avoidant and emotional regulatory coping.

Experiential avoidance tendency is associated with the expression of both negative affect [13] and self-reporting of depressive symptomology [37]. Age also has been shown to relate to coping [38]. Therefore, affect and age will be investigated alongside coping function. It is hypothesised that higher experiential avoidance, avoidance and emotional regulatory coping will be associated with increased negative affect and decreased positive affect. Conversely, older age will be associated with increased approach and reappraisal coping.

In its original conceptualisation, the EAM focuses exclusively on ‘self-injurious behavior that occurs in the absence of any intent to die’ [8], arguing that the distinction between non-suicidal self-injury and behaviour that involved any intent to die is important, ‘given evidence of differences in the functions’ of non-suicidal self-injury and suicide attempts [8]. Other conceptualisations consider suicidal behaviour as an ‘extreme form of emotional avoidance’ [39]. Indeed, the notion of suicidality as an expression of experiential avoidance is arguably consistent with Baumeister’s [40] depiction of suicide as escape from aversive self-evaluation, Shneidman’s [41] description of ‘egression’ and the drive to escape from an unbearable situation outlined in the Cry of Pain [42]. As such, experiential avoidance may be an important transdiagnostic tendency in a pathway to suicidality [43].

Recent research evidence indicates that experiential avoidance may be a key psychological variable in suicidality (suicidal ideation) and, moreover, that addressing experiential avoidance therapeutically may contribute to a reduction in ideation in suicidal individuals, independently of decreased hopelessness and depressive symptomology [44]. Moreover, reduced distress tolerance [45], increased cognitive rigidity [46], emotion regulation and avoidance focused coping [47] are relate to both experiential avoidance and suicidality. Given that 1) an emerging body of evidence suggests that experiential avoidance is a key construct in self-harm enacted in both the presence and absence of suicidal intent and, 2) taxometric analyses indicate that non-suicidal self-injury does not represent a discrete typology [7], the current study assess the relationship between experiential avoidance, coping and the recency and frequency of self-harm regardless of suicidal intent (e.g. including self-harm with mixed intent, suicidal intent, ambivalence).

## Methods

### Participants

One thousand, three hundred and thirty two (1332) adult participants took part in the study. Participants varied in age between 16 and 69 Years (Mean: 19.57, ±6.22). The majority of the sample was female (75.2%). However it is important to note the omission of demographic information for 194 participants for gender (3.2%, *n* = 42 ‘prefer not to say’; 11.4%, *n* = 152 missing), 151 for age (11.3%). Ethnicity demographics were not captured; however country of completion was assessed. Over forty percent (41.7%) of participants were based in North America, .3% South America, .8% Asia, 6.3% Australasia, .1% Africa and 50.8% Europe (data were missing for one participant).

### Design and Procedure

All data were collected via a cross-sectional, community-based survey, administered online. The study was advertised across online platforms/forums (e.g., Twitter; Reddit etc.) via e-mail listings (e.g., Self injury Support UK) and the School of Psychology, Research Participation Scheme. Where applicable, first year undergraduate students received partial course credit for their participation. In line with ethical considerations regarding informed consent, the exclusion criteria prohibited those aged under 16 years from participation. Recruitment was not topic blind; the study was advertised as, ‘part of an on-going project investigating coping function and self-harmful behaviours’. All advertising materials highlighted that we were ‘recruiting participants who have never self-harmed, as well as those who have.’

The work received ethical approval from the University of Nottingham, School of Psychology Ethics Committee [Ref. 335R]. All participants provided written informed consent.

### Measures

#### Demographic factors

Age, gender and continent of residency demographics were collected.

#### Experiential avoidance

Acceptance and Action Questionnaire-II (AAQII) [48] is a 7-item self-report measure which considers an individual’s willingness and ability to stay in contact with aversive internal experiences (e.g., ‘Emotions cause problems in my life’, ‘My painful memories prevent me from having a fulfilling life’, ‘My painful experiences and memories make it difficult for me to live a life that I would value’). Item scores are summed; higher scores indicate higher endorsement of experiential avoidance tendencies. The AAQ-II has demonstrated adequate psychometric properties (Bond et al., 2011). Internal consistency for the AAQ-II in the present study was excellent (α = .937).

#### Coping

Functional Dimensions of Coping scale (FDC) [21] is a three-stage measure, capturing both qualitatively and quantitatively how people attempt to cope with situations they find stressful or distressing. The measure assesses what an individual believes their engagement in specific behaviours will achieve. Parts one and two allow the participant to describe (1) a stressful event they have experienced (in the previous 3 months) and (2) the coping responses (i.e., cognitions, behaviours) they used to help them deal with this (free response). A 16-item series of Likert measures permit indication of the perceived coping function of these coping responses. Item scores are summed; higher scores indicate higher endorsement of coping function.

The measure pertains to four dimensions of coping: emotional regulation (dealing with the emotional consequences of a problem, e.g., ‘To what extent did this/these activities allow you to manage the distress and upset caused by the event?’), approach functions (dealing directly with the problem, e.g., ‘To what extent did this/these activities allow you to understand something of the nature of the problem, from which you could attempt to deal directly with it?’), reappraisal (readdressing and reinterpreting the meaning of a situation, e.g., ‘To what extent did this/these activities allow you to step back and look at the problem, in a different way, such that it seemed better?’) and avoidance functions (allowing the individual to ignore the existence of the situation, e.g., ‘To what extent did this/these activities distract you from thinking about the problem?’).

It is important to note that the FDC measure deliberately does not restrict focus exclusively to a single coping response. The multi-dimensional nature of the scale allows for easy accommodation of the simultaneous, multiple functions typical of self-harm [17].The measure exhibits good internal reliability and construct validity. Internal consistency for FDC subscales in the present study was good (approach, α = .863; avoidance, α = .777; emotion regulation, α = .794; reappraisal, α = .875)

#### Affect

Positive And Negative Affect Scale (PANAS) [49] is a 20-item measure of general mood. Ten items pertain to positive emotional states (e.g., excited; determined; proud) and 10 describe negative states (e.g., distressed; hostile; irritable). Participants were instructed to rate momentary affect. The measure exhibited excellent internal consistency for both positive (α = .862) and negative (α = .926) subscales in the current sample.

#### Self-harm

Section one of the Inventory of Statements about Self-Injury (ISAS) [50,51] was administered to assess lifetime frequency of 12 behaviours (i.e., banging/ hitting self; biting; burning; carving; cutting; wound picking; needle-sticking; pinching; hair pulling; rubbing skin against rough surfaces; severe scratching; swallowing chemicals). The measure includes an “other” category, allowing free report of self-harm behaviours. In line with the phrasing of the original measure, 662 (49.7%) participants indicated behaviours performed ‘intentionally (i.e., on purpose) and without suicidal intent’. Six hundred and seventy participants (50.3%) completed the item considering self-harm irrespective of suicidal intent, or lack thereof. These groups were combined prior to analysis, given that all those who self-harm fall along a continuum of intent [7,52].

#### Recency of last self-harm episode

The Inventory of Statements about Self-Injury (ISAS) [50,51] captures information regarding when the most recent episode of self-harm occurred (section one, question 3). Date stamping for survey completion allowed for a recency variable to be computed, representing the number of days since last self-harm episode. Where exact date information was not available, a metric was generated to ensure that standardised rules were applied across participants. For example, in instances where just a month was indicated (e.g., ‘August’), recency was computed from the 15^th^ of that month (i.e., 15^th^ August).

### Data analysis

Data were analysed using SPSS V21 for Windows. Experiential avoidance, approach, avoidance, emotion regulation and reappraisal were normally distributed. Recency and lifetime frequency demonstrated significant skew. It is also important to note the markedly unequal group sizes when considering lifetime dichotomised engagement with self-harm. Therefore, in all analyses non-parametric tests were performed. In all instances of missing data, cases were excluded pairwise.

A series of Spearman Rho correlations were conducted to test whether coping functions and experiential avoidance were associated with age and affect. As significant relationships were observed, age and affect were included in subsequent analyses and multivariate analyses were adjusted for their effect.

In order to compare the endorsement of experiential avoidance and coping functions between those with and without a lifetime history of self-harm a series of binary logistic regressions were conducted. Experiential avoidance and coping functions were then entered into multivariate binary logistic regressions, to explore effects when adjusting for age and affect.

A series of univariate negative binomial regressions were conducted to explore whether there is a relationship between experiential avoidance, coping function and the recency of self-harm. The influence of age and affect on self-harm recency were also explored. Experiential avoidance and coping functions were then entered into multivariate negative binomial regression, to explore effects when adjusting for age and affect. Finally, the effect of experiential avoidance, coping function, age and affect on lifetime frequency of self-harm was assessed by a series of univariate negative binomial regressions before these variables were entered into multivariate analysis. Negative binomial regressions were selected as, in addition to being suited to count data, this analytic approach models over-dispersion. Negative binomial regression models therefore provided a better fit to the data than scale weighted Poisson regressions ($1/\frac{\chi^{2}}{n-p}$, where *p* is the number of known model parameters).

## Results

### Preliminary analyses

Of the 1332 individuals in the sample, 88.1(%) reported lifetime self-harm. Of those who had ever self-harmed, 115 (10.1%) participants had self-harmed on the day of the study, 45.3% (cumulative percentage) had within the last week, 54.2% the last fortnight, 65.6% in the last month (31 days). The number of days since last episode of self-harm ranged from 0 to 12,775 days (35 years).

The majority of the sample who reported self-harm histories reported high frequencies of behaviour engagement. Nearly one third (29.8%) of participants reported more than 500 self-harm episodes during their lifetime (501–1,000 episodes, 12.9%; 1,001–5,000, 12.9%; >5,000 episodes, 4.0%), with a further 44.5% of participants reporting having self-harmed 101–500 times. Around twenty-five percent (25.8%) of participants reported engaging 100 times or less (1–5 episodes, 1.7%; 6–50 episodes, 11.2%; 51–100 episodes, 12.9%). Self-cutting was the most commonly reported method of self-harm (see Table 2).

**Table 2:** Methods of self-harm reported by participants with a lifetime history of self-harm, *n* = 1173.

Self-harm method (lifetime)	*n*	(%)
Cutting	1071	(91.3)
Interfering with wound healing	883	(75.3)
Banging or hitting self	864	(73.7)
Severe scratching	850	(72.5)
Pinching skin against a rough surface	723	(61.6)
Biting	671	(57.2)
Burning	645	(55.0)
Pulling hair	574	(48.9)
Carving	490	(41.8)
Sticking self with needles	428	(36.5)
Rubbing	420	(35.8)
Swallowing dangerous substances	409	(34.9)
Other	133	(11.3)

*Note*. Participants indicated self-harm behaviours which they had ever engaged in—therefore, many participants are indicated in more than one group. ‘Other’ self-harm methods include medication overdoses, strangling, hanging, asphyxiation, choking, falling/ jumping from high places/ onto hard surfaces, etc.

Thirteen participants (<1%) reported extremely high frequency estimates (e.g., >10^9). On examination of the ISAS responses it appeared that these participants had entered predominantly large, rounded figures on all indicated behaviours. The figures were therefore taken to indicate frequent engagement (e.g., ‘thousands of times’) rather than a literal count and lifetime frequency was capped at 50,000 for subsequent analyses. This did not affect the sample median (= 198) or IQR (= 475).

The relationships between experiential avoidance, coping functions and demographic/ contextual factors across both conditions were explored (see Table 3). Spearman’s correlations indicated that older age was related to lower levels of experiential avoidance and avoidance coping and increased levels of approach, emotion regulation and reappraisal coping functions. The same pattern of relationships were observed for positive affect. Conversely, increased negative affect was related to increased experiential avoidance and avoidance coping and decreased endorsement of approach, emotion regulation and reappraisal coping functions Therefore, after conducting univariate analyses all variables were entered into multivariate regressions, adjusting for age and affect.

**Table 3:** Spearman Rho correlation analyses investigating the associations between experiential avoidance, coping function, age and affect (*N* = 1332).

	1	2	3	4	5	6^^^	7
Experiential avoidance^a^							
Approach^b^	-.399***						
Avoidance^b^	.227***	-.222***					
Emotion regulation^b^	-.081**	.241***	.340***				
Reappraisal^b^	-.405***	.813***	-.128***	.256***			
Negative affect	.690***	-.335***	.225***	-.089**	-.360***		
Positive affect	-.421***	.329***	-.132***	.076*	.370***	-.252***	
Age^†^	-.419***	.310***	-.193***	.071*	.288***	-.428***	.220***
Median (IQR)	39.00 (15.00)	5.00 (10.00)	14.00 (11.00)	12.00 (7.00)	6.00 (12.00)	29.00 (19.00)	18.00 (8.00)

Note.

^a^Experiential Avoidance, as measured by the AAQ-II.

^b^Functional coping dynamics, as measured by the FDC scale.

* denotes significance at *p* <.05,

** denotes significance at *p* <.01,

*** denotes significance at *p* <.001, **bold** typeface indicates significant relationship when Bonferroni corrected (p <.006),

^*n* = 1270,

^†^*n* = 1181, two- tailed.

### Does experiential avoidance and coping differ between individuals with and without a lifetime history of self-harm?

Results of a series of univariate binary logistic regressions indicate that those who have ever engaged in self-harm (*n* = 1173) showed significantly higher levels of experiential avoidance than those with no self-harm history (*n* = 159) (*β =* 1.193, 95% CI: 1.166–1.220, *p* <.001. Model χ^2^ (1) = 403.713, *p* <.001). Further, differences in coping function were also apparent; those with a lifetime history of self-harm were significantly more likely to endorse avoidance (*β =* 1.073, 95% CI: 1.047–1.100, *p* <.001. Model χ^2^ (1) = 32.010, *p* <.001) coping than those who have never self-harmed. Those with a lifetime history of self-harm were also significantly less likely than those who have never self-harmed to endorse approach (*β =* .854, 95% CI: .831– .876, *p* <.001. Model χ^2^ (1) = 155.462, *p* <.001), reappraisal (*β* = .884, 95% CI: .865 –.903, *p* <.001. Model χ^2^ (1) = 141.386, *p* <.001) and emotion regulation coping (*β =* .936, 95% CI: .902 –.972, *p* <.001. Model χ^2^ (1) = 12.806, *p* <.001) in response to a stressor.

Experiential avoidance and coping variables were then entered into a multivariate analysis to explore effects when adjusting for age and affect. Negative affect, age and experiential remain significant (see Table 4).

**Table 4:** Multivariate binary logistic regression exploring whether functional coping dynamics and experiential avoidance predict lifetime history of self-harm, when adjusting for age and affect (*n* = 1173).

	OR	95% CI for OR		*p*
		Lower	Higher	
Negative affect	1.050	1.012	1.090	.010**
Positive affect	1.000	.999	1.000	.234
Age	.950	.921	.979	<.001***
Experiential avoidance^a^	1.137	1.101	1.174	<.001***
Approach^b^	.959	.902	1.020	.186
Avoidance^b^	.976	.933	.1020	.277
Emotion regulation^b^	1.059	.990	1.134	.097
Reappraisal^b^	.973	.926	1.023	.291

*R*^2^ = .288 (Cox & Snell), .555 (Nagelkerke). Model χ^2^ (8) = 384.058, *p* <.001.

Note.

^a^Experiential Avoidance, as measured by the AAQ-II.

^b^Functional coping dynamics, as measured by the FDC scale.

* denotes significance at *p* <.05,

** denotes significance at *p* <.01,

*** denotes significance at *p* <.001.

### Is there a relationship between experiential avoidance, coping function and the recency of self-harm?

A series of univariate negative binomial regressions were conducted to explore whether experiential avoidance and coping functions predicted self-harm recency (see Table 5). The effects of age and affect were also explored. Experiential avoidance significantly predicted the recency of self-harm engagement; the incident rate for number of days since last episode of self-harm decreases by 11.9% for every unit increase in experiential avoidance tendency. Therefore, experiential avoidance was related to increased risk of recent engagement. Increased avoidance was also associated with more recent self-harm; the incident rate for number of days since last episode of self-harm decreases by 6.9% for every unit increase in avoidance coping.

**Table 5:** A series of univariate negative binomial regression exploring whether functional coping dynamics and experiential avoidance predict self-harm recency (*n* = 1173).

	IRR	95% CI for IRR		*p*
		Lower	Higher	
Experiential avoidance^a^	.894	.881	.907	<.001***
Model, χ^2^(1) = 215.642, *p* < .001				
Approach^b^	1.151	1.127	1.176	<.001***
Model, χ^2^(1) = 170.323, *p* < .001				
Avoidance^b^	.935	.918	.952	<.001***
Model, χ^2^(1) = 53.767, *p* < .001				
Emotion Regulation^b^	1.069	1.037	1.102	<.001***
Model, χ^2^(1) = 17.476, *p* < .001				
Reappraisal^b^	1.146	1.126	1.166	<.001***
Model, χ^2^(1) = 246.118, *p* < .001				
Negative affect	.921	.912	.930	*<* .001***
Model, χ^2^(1) = 241.089, *p* < .001				
Positive affect	.999	.999	1.000	*<* .001***
Model, χ^2^(1) = 9.935, *p* = .002				
Age	1.218	1.179	1.258	*<* .001***
Model, χ^2^(1) = 260.304, *p* < .001				

*Note*. IRR; Incident Rate Ratio, coefficients from negative binomial regressions are expressed on a log scale, therefore the table presents the exponent of the regression coefficients.

^a^Experiential Avoidance, as measured by the AAQ-II.

^b^Functional coping dynamics, as measured by the FDC scale.

* denotes significance at *p* <.05,

** denotes significance at *p* <.01,

*** denotes significance at *p* <.001.

Approach, reappraisal and emotion regulation coping were related to an increased period since last episode self-harm. The incident rate for number of days since last episode of self-harm increased by 15.1%, 14.6% and 6.9% for every unit increase in approach, reappraisal and emotion regulation coping respectively.

Increased in the reporting of both positive and negative affect were predictive of more recent self-harm. Age was also predictive of self-harm recency; the incident rate for number of days since last episode of self-harm increased by 21.8% for every year increase in age.

Experiential avoidance, coping functions, age and affect were then entered into a multivariate negative binomial regression to explore the whether coping functions and experiential avoidance predict self-harm recency, when adjusting for age and affect. Negative affect, age and reappraisal coping remained significantly predictive of the recency of self-harm engagement. Here, the incident rate for number of days since last episode of self-harm increased by 8.3% for every unit increase in reappraisal coping (see Table 6). Both reappraisal and older age were related to a decreased risk of recent self-harm. Increased negative affect was related to more recent self-harm. In multivariate analysis, neither experiential avoidance nor approach, avoidance or emotion regulation coping functions were significantly predictive of the recency of self-harm.

**Table 6:** Multivariate negative binomial regression exploring whether functional coping dynamics and experiential avoidance predict self-harm recency, when adjusting for age and affect (*n* = 1173).

	IRR	95% CI for IRR		*p*
		Lower	Upper	
Negative affect	.964	.952	.977	<.001***
Positive affect	.999	.999	1.000	.004**
Age	1.152	1.113	1.193	<.001***
Experiential avoidance^a^	.991	.972	1.009	.322
Approach^b^	.980	.948	1.014	.250
Avoidance^b^	1.012	.994	1.031	.190
Emotion regulation^b^	.990	.962	1.020	.514
Reappraisal^b^	1.083	1.053	1.115	<.001***

Model, χ^2^(8) = 437.341, p <.001.

*Note*. IRR; Incident Rate Ratio, coefficients from negative binomial regressions are expressed on a log scale, therefore the table presents the exponent of the regression coefficients.

^a^Experiential Avoidance, as measured by the AAQ-II.

^b^Functional coping dynamics, as measured by the FDC scale.

* denotes significance at *p* <.05,

** denotes significance at *p* <.01,

*** denotes significance at *p* <.001.

### Is there a relationship between experiential avoidance, coping function and the lifetime frequency of self-harm?

A series of univariate negative binomial regressions were conducted to explore whether, in those with a history of self-harm, experiential avoidance and coping functions predicted lifetime frequency of engagement. The effects of age and affect were also explored. Experiential avoidance significantly predicted the frequency of self-harm engagement; the incident rate for lifetime frequency of self-harm increases by 4.6% for every unit increase in experiential avoidance tendency (IRR = 1.046, *p* = .001, 95% CI: 1.034–1.057. Model, χ^2^(1) = 54.094, *p<* .001). Therefore EA related to increased risk of higher frequency self-harm. Coping functions were also predictive of self-harm. Increased avoidance (IRR = 1.028, *p<* .001, 95% CI: 1.015–1.042. Model, χ ^2^(1) = 17.377, *p<* .001) was also associated with higher frequency self-harm engagement. The incident rate for number of lifetime episode of self-harm increases by 2.8% for every unit increase in functionally avoidant coping. Conversely, increases in both approach (IRR = .943, *p<* .001, 95% CI: .928 –.958. Model, χ^2^(1) = 45.947, *p<* .001) and reappraisal coping (IRR = .959, *p<* .001, 95% CI: .947 –.972. Model, χ^2^(1) = 34.674, *p<* .001) were associated with a decreased likelihood of high frequency self-harm. For every unit increase in approach coping, the incident rate for number of lifetime episodes of self-harm decreased by 6.0%; for reappraisal coping the decrease was 4.2%. Emotion regulation coping was not predictive of lifetime frequency of self-harm (IRR = 1.011, *p* = .236, 95% CI: .993–1.030. Model, χ^2^(1) = 1.394, *p* = .238).

Age (IRR = 1.030, *p* = .040, 95% CI: 1.001–1.060. Model, χ^2^(1) = 4.530, *p =* .033) and positive affect (IRR = 1.000, *p* = .257, 95% CI: 1.000–1.000. Model, χ^2^(1) = 1.327, *p* = .249) were not predictive of lifetime frequency of self-harm.

Negative affect significantly predicted the frequency of self-harm engagement; the incident rate for lifetime frequency of self-harm increases by 3.6% for every unit increase in negative affect (IRR = 1.036, *p<* .001, 95% CI: 1.026–1.046. Model, χ^2^(1) = 47.041, *p<* .001). Therefore negative affect related to increased risk of higher frequency self-harm.

The variables were then entered into a multivariate negative binomial regression in order to account for the effect of age and affect. In multivariate analysis, adjusting for age and affect, approach coping remains significantly predictive of lifetime frequency of self-harm. The incident rate for number of lifetime episode of self-harm decreased by 4.1% for every unit increase in approach coping (see Table 7).

**Table 7:** Multivariate negative binomial regression exploring whether functional coping dynamics and experiential avoidance predict self-harm lifetime frequency, when adjusting for age and affect (*n* = 1173).

	IRR	95% CI for IRR		*p*
		Lower	Upper	
Negative affect	1.031	1.017	1.045	<.001**
Positive affect	1.000	1.000	1.000	.250
Age	1.082	1.049	1.116	<.001**
Experiential avoidance^a^	1.009	.991	1.028	.327
Approach^b^	.960	.929	.991	.013*
Avoidance^b^	1.004	.986	1.023	.661
Emotion regulation^b^	1.023	.998	1.048	.067
Reappraisal^b^	.997	.973	1.023	.832

Model, χ^2^(8) = 88.017, *p* <.001

*Note*. IRR; Incident Rate Ratio, coefficients from negative binomial regressions are expressed on a log scale, therefore the table presents the exponent of the regression coefficients.

^a^Experiential Avoidance, as measured by the AAQ-II.

^b^Functional coping dynamics, as measured by the FDC scale.

* denotes significance at *p* <.05,

** denotes significance at *p* <.01,

*** denotes significance at *p* <.001.

## Discussion

The findings support the hypothesis that those who have engaged in self-harm show significantly higher endorsement of experiential avoidance tendencies, than those who have never self-harmed. This remains the case when experiential avoidance and coping functions are entered into multivariate analyses, adjusted for age and affect. As predicted, differences in the endorsement of coping functions were also observed., with those with a lifetime history of self-harm being significantly more likely to endorse avoidance coping, in univariate analysis. However, contrary to our hypotheses, those with a history of self-harm were lower in emotion regulation coping than those who have never self-harmed. Differences were also apparent in approach and reappraisal coping; those with a lifetime history of self-harm were significantly less likely than those who have never self-harmed to endorse approach coping or reappraisal coping functions, in response to a stressor. Taken together, these findings suggest that the EAM [8] is also a potentially useful theoretical framework when considering self-harm irrespective of suicidal intent, or lack thereof. That is to say, experiential avoidance may be an important factor psychological in self-harm of mixed intent, suicidal intent, or enacted with ambivalent intent, as well as non-suicidal self-injury, as outlined in the EAM. However, the observed effect sizes were small, indicating that other variables may be central in explaining both the initiation and continuation of self-harm behaviours.

More recent self-harm was, in univariate analysis, associated with lower endorsement of approach, reappraisal and emotional regulation coping and also higher levels of both avoidance coping and experiential avoidance. In multivariate analyses, adjusted for age and affect, reappraisal coping remained significantly predictive of the recency of self-harm; reappraisal related to a decreased risk of recent engagement.

Considering the lifetime frequency of self-harm, in univariate analysis, increased experiential avoidance was significantly predictive of higher volume of self-harm engagement. While statistically significant, this effect was small. Considering the relative magnitudes of effects, the results of this study indicate that experiential avoidance may be more useful in explaining the recency of self-harm than the lifetime frequency of behaviour engagement. Increased avoidance was also associated with higher frequency self-harm. Conversely, increases in both approach and reappraisal coping were associated with a decreased likelihood of high frequency self-harm. Emotion regulation coping was not predictive of self-harm frequency. When coping functions and experiential avoidance were entered into multivariate analysis, adjusted for age and affect, approach coping remains significantly predictive of lifetime frequency of self-harm. Increased endorsement of approach coping function was predictive of decreased lifetime frequency of harming.

While these findings offer a novel contribution to the literature it is important that they are interpreted within the context of their limitations; while the Inventory of Statements About Self-Injury [50,51] instructs participants to ‘estimate the number of times in your life you have intentionally (i.e., on purpose) performed each type of self-harm (e.g., 0, 10, 100, 500)’, participants may be unclear as to whether they are to report the number of episodes of self-harm, or the number of discrete injuries. Interpreting the instructions as the number of episodes may lead to marked underestimations in lifetime frequency. Further to this, those with higher volumes of self-harm may round their frequencies for each behaviour to the nearest 10, or 100 (a tendency which may have been amplified by the measure’s inclusion of exclusively rounded numbers in the examples given), whereas those with fewer incidences in total may report more accurately. Some participants entered predominantly very large, rounded figures on all indicated behaviours. The figures were taken to indicate frequent engagement (e.g., ‘thousands of times’) rather than a literal count and lifetime frequency was capped at 50,000, given the spread of the data. While this affected less than 1% of participants, and did not affect the sample Mdn or IQR, this interpretation of the measure and pre-analysis screening should be noted. Finally, recency of engagement may also have influenced lifetime frequency findings, as well as an individual’s ability to accurately report on an event; accurate recall of more distal events may be hampered by memory biases.

When considering the relationship observed between emotion regulation coping and self-harm, it is pertinent to recognise the potential influence of the measure elected to assess coping dynamics. While the Functional Dimension of Coping [21] scale outlines emotion regulation as ‘behaviours that the person believes will allow them to deal with the emotional consequences of the stressful encounter’ (section 1, page 1), in line with conceptualisations outlined in the EAM [8], arguably a number of the scale items appear to relate more readily to the efficacy of these efforts, rather than the motivational and functional dynamics underpinning the coping effort per se (e.g., ‘To what extent did this/these activities allow you to manage the distress and upset caused by the event?’, ‘To what extent did this/these activities allow you to handle any anxiety caused by the event?’). Therefore, it may be that while individuals who self-harm are motivated to deal with aversive emotional outcomes of stressful/distressing events, they are low in endorsing the emotion regulatory items, due to the perceived inefficacy of their actions. Given the established literature linking self-harm to difficulties in emotion regulation [53] such explanations could explain the pattern of results observed; specifically, why our results indicate that emotion regulation coping was lower in those with a lifetime history of self-harm and also why increased emotion regulation coping was related to an increased period since last episode self-harm. Further support for this suggestion may also be taken from the small association observed between the level of endorsement of experiential avoidance and emotion regulation coping functions. The current study did not capture engagement in alternative problematic behaviours (e.g., substance misuse). Given that such behaviours may serve as an effective short-term strategy for experiential manipulation [10], further work would benefit from a more nuanced sample characterisation, including the assessment of divergent manifestations of emotion regulation.

To the best of our knowledge this is the first study to examine coping functions, rather than coping styles, in relation to lifetime self-harm and self-harm recency and frequency. Taken together, results indicate that reappraisal and approach coping functions may be important protective factors in self-harm. After adjusting for affect, in multivariate analyses, higher levels of reappraisal coping were associated with less recent self-harm and higher levels of approach coping with lower life-time frequency of self-harm. Unlike many static risk factors, functional coping is by nature dynamic. Transactional accounts consider coping responses to be context and time dependant—rather than viewing coping behaviours and cognitions as dispositional styles. Thus, inherent to functional coping is the notion of change; coping is modifiable. This has implications for clinical practice and suggests coping which an individual believes allows them to 1) directly confront problems faced (approach), and 2) readdress the problems faced and reinterpret the meaning of a situation (reappraisal) [21] may be particularly important intervention targets. Results of the multivariate analysis indicate that experiential avoidance can differentiate those with and without a lifetime history of self-harm. In univariate analysis, experiential avoidance was also predictive of recency and lifetime frequency. This suggest that experiential avoidance may be an important transdiagnostic processes in self-harm, regardless of suicidal intent.

In the current study, approach and reappraisal coping functions were highly correlated. This has implications for the conceptualisation of coping. While Cox and Ferguson [21,25] outline four related, yet qualitatively distinct, coping dimensions, alternative conceptual frameworks delineate coping processes into divergent dimensions [28,29]. As Ferguson and Cox [21] note, the functional dimensions outlined within the FDC may be subsumed within an alternative, more parsimonious account. When considering the implications of the current results it may be particular pertinent to note Holahan & Moos’ [33,34] conceptualisation of transactional coping. Here, two functional coping dimensions (approach—avoidance) are outlined, with reappraisal-type items subsumed within the problem-focused, approach function. This may account for the large effect size observed. Within this framework, a positive correlation between emotion regulation and approach coping would also be anticipated. While such a relationship was observed, the effect size was small. However, this may be explained by the phrasing of the emotion regulation items, as outlined above.

As hypothesised, increased endorsement of experiential avoidance and avoidance coping functions were associated with increased negative affect. Reduced approach, reappraisal and contrary to expectations, emotional regulation coping functions were associated with increased negative affect. A pattern of relationships with converse directionality is observed in positive affect. In line with the hypothesis, older age was related to increased approach and reappraisal coping as well as decreased experiential avoidance tendency and functionally avoidance coping. Older participants also reported significantly higher levels of emotion regulation coping.

While mixed intent, or varying degrees of ambivalence are commonly reported in self-harm [7,52], it is important to note that the proportion of participants in this study who endorse only non-suicidal behaviour is unknown. An extension of this work would therefore be to explore the distribution of intent (e.g., how many participants endorse only self-harm with no suicidal intent; how many participants endorse suicidality etc.), as well as the intent histories of participants (e.g., what proportion of respondents have a lifetime history of suicidal behaviours, but had recently engaged in only non-suicidal self-harm).

When interpreting these findings it is important to note the nature of the sample. A key advantage of the study is the large, non-clinical sample. The majority of empirical support for the EAM [8] and the model’s theoretical foundations, are derived from the literature focused on self-harm exclusively in the context of Borderline Personality Disorder (BPD). As the model was developed to apply at a general level across various populations, and given the increasing concern regarding self-harm in non-clinical populations [54,55], it is important to extend current knowledge by exploring coping and emotional avoidance dynamics, and testing the models assertions, in broader community samples.

A large majority of participants had a lifetime history of self-harm (88.1%) which is markedly higher than rates typically reported for self-harm [56,57] and NSSI [58] in non-clinical research. This is a result of a deliberate recruitment strategy in order to achieve a large enough sample of people with a history of self-harm to meaningfully explore recency and frequency, with adequate power and without collapsing data into arbitrarily defined categories (e.g. 6 months, 12 months) [36].

Despite efforts to recruit a diverse sample, the sample achieved contained a large proportion of recent, high frequency self-harm; 65.3% had self-harmed in the last month. Therefore, while considering ‘lifetime’ engagement, for many this was very recent, or in fact current, engagement. This is important when considering ‘recovery’ and the dynamics that may underpin it: while the results suggest that coping dynamics and levels of emotional avoidance differ dependant on whether an individual has ever engaged in self-harm, caution is advised in the strength given to these claims. It is less clear if, and how, the relationships between variables may differ when considering more long-term resolution of self-harm engagement. Future work should seek to recruit a more varied sample in terms of the volume and recency of behaviour engagement.

### Limitations

The primary limitation of the work is the cross-sectional nature of the study, precluding the ability to draw conclusions regarding causality. While the study offers a preliminary consideration of coping function and experiential avoidance in relation to the recency and frequency of self-harm, longitudinal research is optimally placed to explore these dynamics more fully. Such research offers the potential to address the current lack of clarity regarding how functional coping dynamics and non-acceptance of internal negative experiences may map to behaviour change. Research exploring coping which an individual believes will 1) allow them to deal directly with a stressor, and/or 2) allow them to reinterpret or construct the problem faced, may be particularly important. Moreover, in addition to insight into temporal dynamics, such investigations would permit the exploration of interactions with proposed moderators, such as distress tolerance [8]. Importantly, prospective work may help partial out psychological context. That is, while self-harm is a symptom of distress it can occur in the context of a range of psychological disorders, including, but not limited to, BPD, anxiety, major depression, substance abuse and oppositional defiant or conduct disorder [59,60]. The ability to adjust for psychological context may be important given the demonstrated relationship between psychopathology and experiential avoidance [61].

The current study considers the recency and frequency of self-harm independently. While this offers novel insight, future research may benefit from the exploration of the combined effects of recency and frequency. For example, equivalent overall lifetime frequency may be reported by an individual who previously self-harmed, yet has not done so for some years and by an individual who currently self-harms often. Arguably, it could be expected that the dynamics underlying these situations may be divergent. The duration of time over which an individual has engaged in self-harm (i.e., time since first self-harm) and any effect this may have on the associations between self-harm, experiential avoidance and coping functions, also warrants future attention.

Although the study recruited a community sample this sample was not representative. A disproportionate number of participants were under 25 years of age (88.9%) and female (74.9%). It would be interesting to test the replicability of findings in an older, or more diverse sample.

Finally, the study is limited by its reliance on mono-method, self-report assessment. The ability to accurately report on internal experiences may be confounded by an individual’s aptitude for accessing states, and willingness to engage with aversive experience. A reluctance, or difficulty in doing so, is likely to be marked in those with heightened tendencies for experiential avoidance. Thus, multimodal assessment may be optimal, with the inclusion of behavioural and/or experimental paradigms adding additional strength to protocols investigating experiential avoidance tendencies [62].

Notwithstanding these limitations, the study offers novel insight into the relationship between self-harm, experiential avoidance and coping function, expanding on the existing literature to consider the recency and frequency of self-harm engagement. Additionally, and for the first time, the study explores these relationships in both non-suicidal self-injury and self-harm irrespective of (suicidal) intent, comparing findings between groups.
