# Correction: Exploring the Relationship between Experiential Avoidance, Coping Functions and the Recency and Frequency of Self-Harm

**DOI:** 10.1371/journal.pone.0192795

**Published:** 2018-02-14

**Authors:** Emma Nielsen, Kapil Sayal, Ellen Townsend

There is an error in the first sentence of the fourth paragraph of the results section. The correct sentence is as follows: The relationships between experiential avoidance, coping functions and demographic/ contextual factors were explored (see Table 3).

The last paragraph of the Limitations section is incorrect. The correct paragraph is as follows: Notwithstanding these limitations, the study offers novel insight into the relationship between self-harm, experiential avoidance and coping function, expanding on the existing literature to consider the recency and frequency of self-harm engagement.

There are errors in the Author Contributions. The correct contributions are: Conceived and designed the experiments: EN KS ET. Performed the experiments: EN. Analyzed the data: EN KS ET. Wrote the paper: EN KS ET.
